# Diagnosis of Gastric Cancer

**Published:** 1881-08

**Authors:** 


Diagnosis of Gastric Cancer.—The large experience and close analysis of symptoms and signs of disease, enabled the late distinguished Professor Trousseau to add a most important aid to the correct diagnosis of cancer of the stomach. As he was himself a victim of the terrible disease, he naturally studied the phenomena connected with it, with great closeness and accuracy, and such was his confidence in the diagnostic, if not pathognomic, value of thumbosis, that upon the occurrence of *phlegnasia dolens* in his own case, he assured his dear friend and medical attendant, that his end was at hand. Should phlegnasia alba dolens occur in either the upper or lower extremity, the diagnosis is rendered certain in gastric disease.

